# The Trajectories of Digital Skills and Their Determinants Among Middle-Aged and Older Koreans

**DOI:** 10.1093/geroni/igaf122.1454

**Published:** 2025-12-31

**Authors:** Miseon Kang, Hyo Jung Lee

**Affiliations:** Symbiotic Life-TECH Institute, Seoul, Republic of Korea; Yonsei University, Seoul, Republic of Korea

## Abstract

The digital divide manifests within and between the people in diverse aspects. This phenomenon became more evident during COVID-19 pandemic, which profoundly accelerated digitalization. In this context, individuals exhibit diverse trajectories in their digital skills, shaped by differing life choices, social positions, and environments. Recognizing this, this study explored distinct patterns of digital skills trajectories among middle-aged and older Koreans and examined the key determinants influencing these trajectories. ‘The Korea Media Panel Survey’ (2018-2021) was used, and the sample included 2,291 middle-aged Koreans (aged 45-64) and 1,528 older Koreans (aged 65 and above). Latent class growth analysis and multinominal logistic regression were employed. The analysis identified five digital skills trajectory types among middle-aged adults: ‘Digitally advanced (50.1%)’, ‘Digitally shrinking (11.8%)’, ‘Digitally transforming (18.0%)’, ‘Digitally adapting (16.0%)’, and ‘Digitally stalled (4.1%)’. Among older adults, three digital skills trajectories were identified: ‘Digitally skilled (5.6%)’, ‘Digitally adapting (26.8%)’, and ‘Digitally dragging (67.6%)’. For middle-aged adults, significant determinants of digital skills trajectory types were age, gender, educational level, employment status, monthly income, residential area, need for cognition, and smartphone ownership. For older adults, age, gender, educational level, monthly income, residential area, marital status, need for cognition, and smartphone ownership predicted the types of digital skills trajectories. These findings highlight the heterogeneity of digital skills trajectories in middle-aged and older adults and underscore the importance of these specific determinants in designing tailored interventions to promote digital skills and competencies for these populations.

